# An Essay on the Philosophy of Medical Science

**Published:** 1845-07

**Authors:** 


### Art. XII.

*An Essay on the Philosophy of Medical Science.* By Elisha Bartlett, m.d. Professor of the Theory and Practice of Medicine in the University of Maryland.—*Philadelphia,* 1844. 8vo, pp. 312.

The purpose of this work is a most laudable one—the advocacy of careful and accurate observation of the phenomena of disease, as the only source of real improvements in medical practice. We think, however, that he has pushed his argument rather to an extreme; and that he does not attach sufficient value to *principles,* as the chief constituents of the *science* of medicine. As we have on two preceding occasions formally treated this subject, vol. V, p. 317, and vol. VI, p. 98, we may refer to the articles in question for a fuller exposition of our own views in regard to it; and shall confine ourselves at present to the examination of a few points, in which we think that Dr. Bartlett has gone wrong.

He assumes *in limine* the following positions:

"1. All physical science consists in ascertained facts, or phenomena, or events; with their relations to other facts, phenomena, or events; the whole classified and arranged.

"2. These facts, phenomena, and events, with their relations, can be ascertained only in one way; and that is, by observation or experience. They cannot be deduced or inferred from any other facts, phenomena, events, or relationships, by any process of reasoning, independent of observation or experience.

"3. A law, or principle, of physical science consists in a rigorous and absolute generalization of these facts, phenomena, events and relationships; and in nothing else. It is identical with the universality of a phenomenon, or the invariableness of a relationship." (p. 3.)

These canons *appear* entirely coincident with the views of the best authorities on the subject, as well as with the dictates of common sense; and yet there lurks in them—we will not call it a fallacy but—a departure from the ordinary acceptation of terms, which lies at the root of that which we deem erroneous in the subsequent part of the work. We shall devote a short space, therefore, to the analysis of their meaning; which is more fully explained in the following quotation:

"The first proposition, that which stands at the head of this chapter, does not require much illustration. Its truth is so manifest, as hardly to admit of any doubt. It would seem almost impossible that there should be any difference of opinion as to its soundness or obscurity in its conception. I believe, nevertheless, it is true, that there has always been, and that there still is, in the minds of most men, and in those of philosophical thinkers, a somewhat imperfect, or confused, apprehension of its doctrines. I do not think that its truth is seen and felt, as it should be, in the simplicity, the purity, and the absoluteness, which belong to it.

The confusion, to which I allude, is this. There seems to be a common feeling, that the facts, phenomena, and events, with their relationships, classified and arranged, constitute, not the entire science, to which they belong, but only the *foundation* of the science. There is a feeling that these facts and relations are to be used as elements, out of which the science is to be built up, or constructed, by what is called *inductive reasoning*. The feeling implies, and the avowed doctrine growing out of it often asserts, that the science *is in this subsequent process of reasoning*, and not in the facts themselves or their relationships. We are constantly told, that the facts are to be used *as materials*, to be sure; that it is not safe to take for our materials anything but facts; that they constitute the *basis* of every science; but after all this, the essential condition and constituent of the science is often placed more in the process of reasoning, as it is called, than in the facts and their relationships. Now, what I wish to insist upon is this; that the science *is in the facts and their relationships, classified and arranged, and in nothing else*. The ascertained facts and their relationships, classified and arranged, constitute, in themselves, and alone, the science and the whole science to which they belong." (pp. 6-8.)

Now Dr. Bartlett has an undoubted right to use the term *science*, or any other, in whatever sense he thinks proper, first defining (as he has here very clearly done) the exact meaning which he attaches to it. Nevertheless we question whether it is well to depart so widely from the received acceptation of the term; connected, as it is, in the minds of philosophers, with an idea so very distinct as that which Bacon and his followers have attached to it. We may best, perhaps, illustrate our own conception of its meaning, and the point wherein we differ from Dr. Bartlett, by analysing one of his own examples. "The whole science of gravitation," he says, (p. 9,) "consists in its phenomena, classified and arranged, and in nothing else." Now on this we remark, in the first place, that the simple collection and classification of phenomena does not necessarily constitute science. We may adopt a wrong principle of classification, and thus be as far from discovering any general principles as if we had made no arrangement at all; this has been most egregiously the case in the various artificial systems of natural history: or, having adopted a right method of classification, we may not be able to grasp the true principles regulating the phenomena, in consequence of the agency of some modifying circumstance, which so disturbs its results, as to prevent their true relation from being seen. This was the case in regard to the influence of the resistance of the air upon falling bodies; which caused the ancient philosophers and their blinded followers to maintain, that the law of gravitation was quite different for light and for heavy bodies,—until they were set right by Galileo, who may be said to be the discoverer of the true law of terrestrial gravitation, as Newton was of the bearing of that law on celestial phenomena.

Now it is in the discovery of such *laws*, that *science* consists; and in proportion as any group of phenomena is included within laws, that are not only general expressions of the phenomena with which we are already acquainted, but are found to be equally applicable to those which are progressively discovered,—so as to give to the philosopher in possession of them the power of *predicting* with certainty the results of new combinations of circumstances,—in that proportion do they constitute a *science*. This power of *prediction*, the most valuable result of the attainment of general laws, seems to us to have been almost completely overlooked by

Dr. Bartlett; and his want of appreciation of the real nature of *scientific* as distinguished from *empirical* knowledge, is obvious to us throughout his essay. Nothing but the collocation and classification of the phenomena is required for the deduction of *empirical* laws; thus by bringing together the number of deaths at various ages, which occur in a given population during a certain time, the *law* of the rate of mortality and the expectation of life can be deduced; or by similar statistical calculations, the proportion of crime to education may be determined. If science consisted of such laws as these, Dr. Bartlett would be perfectly correct in his appreciation of it; but a very little consideration must make it apparent, that—however useful in themselves—they are of a character entirely different from those general laws, which it is the object of the philosopher to trace out amongst the phenomena of nature. Let us contrast, for example, the law of gravitation with that regulating the rate of mortality in a given district. The former enables us to measure the force which, under all circumstances whatever, will tend to draw together two bodies of known weights, and to predict *with absolute certainty* the result of the conjoint operation of this force with others. It admits of no exceptions; its comprehensiveness is only equalled by its simplicity; and it connects, like the secret clue of a labyrinth, all the phenomena which bear the slightest relation to it. But a table of mortality expresses only *probabilities.* It assumes the experience of the past as the guide to the future, but only so far as the same causes may be presumed to be in action; its application is consequently of the most limited nature, being liable to be contravened by any change in the almost numberless influences which may bear upon it; and there is nothing like a common relation,—save that of the fact of *death*—binding together the different phenomena. For the discovery of truth, these empirical formulæ are often of great value; but we must not stop short in them, and plume ourselves with the idea of having arrived at our end, when we are, in fact, only in the very beginning.

We could scarcely find a better illustration of the difference between empirical laws and real generalizations, than the comparison of the laws of planetary motion, discovered by Kepler, and the fundamental laws of motion enunciated by Newton. Kepler, after almost an infinity of guesses,—the mingled ingenuity and absurdity of which we can scarcely help admiring and pitying—at last stumbled, by a wonderful piece of good fortune, upon those laws which pass by his name. He perceived that the inequalities in the rate of the planet Mars might be accounted for by the supposition, that its "radius vector passes over equal areas of its elliptical orbit in equal times." This was (for the time) an empirical formula, which perfectly well expressed the inequalities in the rate of motion of the planet Mars; but which might, or might not, be equally applicable to those of other planets. It was found, on examination, to be thus applicable; and this was an important step in true generalization. Still, however, the formula was so far empirical, that no reason could be assigned *why* it should prevail elsewhere; nor could it be predicted whether, in the case of a newly-discovered planet, the same rule would apply. It was reserved for Newton, however, to give to this formula (and also to the other on which Kepler had been fortunate enough to stumble, viz. "that the squares of the times of revolution are as the cubes of the distances,") the dignity of a real *law;* by showing that it necessarily results from the combination of the simple principle of gravitation with the laws of motion; and that, as *these* are of universal operation, *it* must govern *every* case of elliptical revolution.

Having said so much upon the general question, we shall not follow Dr. Bartlett through the details of his inquiry into the value of hypotheses in physical science. Most truly does he remark, that all approved and stable generalizations in science are based upon an extended foundation of facts; and that, however striking and seductive such generalizations may be, they have no claim to be received as certainties, until they have been shown to possess such a foundation. But in his zeal against hypotheses, he has altogether forgotten that most, if not all, of those scientific principles, on which we feel that we can securely rest, were first brought forward in that very form; and that it was only after the long and patient application of tests, by which their truth or falsity might be established, that they have acquired their present character. Thus the law of universal gravitation was long a hypothesis floating in the vast mind of Newton. So far as related to the fall of small bodies towards the earth, it had been determined by his predecessors; but by sagacious reasoning he was led to the idea, that the moon might be held in her orbit round the earth by a similar attractive force. Now this might be called (and was in fact considered at the time) a most daring and fanciful *hypothesis.* On first attempting to verify it, Newton was baffled by errors in the data on which he worked; and finding that the conclusions of experience did not correspond with those of his theory, he put the latter aside, in hope, as it subsequently proved, that more accurate data might be found to afford the required correspondence. This was the case some years afterwards; the length of a degree was ascertained to be sixty-nine and a half miles instead of sixty; the measure of the diameter of the earth was consequently increased; and the calculated deflection of the moon from a straight course in a given time, was found to correspond so exactly with her real departure, that the *hypothesis* became a *law,* so far at least as the moon was concerned. Its extension to the other planets, and to comets, was the work of Newton himself; but its grasp of a far more gigantic range of phenomena,—the mutual revolutions of the double and triple stars,—has been the result of the labours of our own contemporaries. Until it could be shown that the principle of mutual attraction operates, not only upon the bodies composing our solar system, but also upon those which make up the stellar universe, its *universality* was a *hypothesis.*

In the same manner it would be easy to show, that the *law* of definite proportions was, in the mind of Dalton, at first a hypothesis, based upon a few facts, and not entitled to any higher dignity, until it had been tested by more extended inquiry. The universality of its application, in organic as well as in inorganic chemistry, is only now being recognized; and in some of its applications, this law must still be regarded as hypothetical.

It appears, then, that the boundary between *hypothesis* and *law* is not by any means so clear as it might seem at first sight; for that every law must be more or less of a hypothesis, until its applicability to all the phenomena, which it can possibly have a share in producing, has been tested. The truly philosophic mind, it has been well remarked, shows itself in the readiness or aptitude for the construction of hypotheses,—in the equal readiness to abandon them, so soon as their instability has been proved,—and in the caution with which they are employed, whilst on their trial.

There is, probably, no science in which this caution is more necessary than in medicine; for there is none in which it is more difficult to frame a really good hypothesis, and none in which the devotion to a bad one is more prejudicial. But *some* hypothesis is absolutely necessary. And we fearlessly assert that the *scientific* physician, whose knowledge of the theory of medicine is applied with even a moderate degree of judgment, is less open to the charge of misuse of hypothesis, than is the *empirical** practitioner, who professes to found his treatment immediately upon experience, and to discard theory altogether. For the latter assumes a sweeping hypothesis as the very basis of his practice; viz., that a plan of treatment which he has found successful in one case will be probably alike successful in another which may present a general similarity in its phenomena; and he puts on one side, as of no account in comparison with what strike him as the leading features of the case, those slighter differences which are, to the more scientific, the most important indications in respect to the real nature of the disease, and the indications for treatment. Thus have we seen a physician of days gone by prescribe bleeding after bleeding for a chlorotic patient, simply because she complained of pain in her side, and her blood presented a buffy coat. Is the practitioner who is aware of the real nature of the buffy coat, the conditions of its formation, the deficiency of red corpuscles, and the influence of iron in producing an increased production of them, to be charged with a dangerous use of hypothesis, because in such a case he prescribes ferruginous medicines instead of venesection? Surely not. The danger is on the side of the practitioner, who *presumes* that a buffy coat and pain in the side are indications of inflammatory action; and who, adopting the lancet as his chief instrument for combating that inflammation, reduces his unfortunate patient to a state of almost absolute anæmia.

Let us take a still more extreme case. The professed empiric, who vaunts his secret remedy as a certain cure for all diseases, proceeds upon the *hypothesis*, (see the advertisements of Messrs. Morison and Moat, hygeists,) that all disorders have one common origin, which his panacea can remove. Whether he believes this hypothesis or not, he causes the public to believe it, and to act upon it. Now we do not see any very wide distinction between *his* method, and that of the routine practitioner, who attributes nine tenths of the diseases which present themselves for his treatment, to some single organ; or who uses for their cure a limited number of remedies, with little regard to any but certain prominent symptoms, upon which his mind rests as the indications of his treatment. Whether the brain, the stomach, the spinal cord, the liver, the spleen, the pancreas, or the kidneys, whether the solids of the body, or its fluids, be in his mind the favorite seat of disease, the case is fundamentally the same; his practice is founded on a *hypothesis*, which attributes a great variety of effects to a common cause; and this hypothesis is the more erroneous, and consequently injurious, as being founded on a very limited and imperfect

* We here use this term in its classical sense.

knowledge of the phenomena to which it applies. Or if, whatever be his ideas in regard to the nature and seat of the disease, the practitioner places his whole trust in a limited number of remedies, and uses these with little discrimination in a great variety of cases, the rationale of his practice (if rationale it can be said to have) is a *hypothesis,* which assumes a similarity in the effects of particular medicines, when there is an apparent correspondence in the symptoms which they are intended to alleviate.

We have now attempted to show that *all* application of remedies to disease must be to a certain extent hypothetical; and if this be granted, we do not think it will be denied that the greater the comprehensiveness of the hypothesis, and the more extended the foundation of ascertained facts on which it is erected, the greater will be the amount of confidence it deserves as a guide in the therapeutic art. The distinction between the scientific and the routine practitioner, therefore, is not in the employment of hypothesis by the former, and the rejection of it by the latter, but in the superior validity of the hypotheses adopted by the former over those assumed by the latter, arising out of the greater accuracy and more extensive range of the observations on which they are based.

Having said so much upon the general questions discussed by Dr. Bartlett, it is the less necessary that we should follow him into the particulars of the second portion of the work, which treats of the Philosophy of Medical Science. As already stated, we differ from him widely as to what constitutes *Science;* and as there is scarcely any part of the Essay which is not imbued with the peculiar and (in our apprehension) erroneous view which he has taken of its character, there is very little with which we can heartily accord. The following passage expresses the chief peculiarity of his doctrine:

> "The feeling has been, and still is, as much, almost since the time of Bacon as before,—that the scieuce is *in the inductive or reasoning* process, superadded to the facts and their relations, more than in these latter themselves. Here, at the commencement of this part of my Essay, I wish to enter my protest against this doctrine in all its forms and modifications. I wish to show that the science of medicine consists *in the phenomena of life, with their relationships, classified and arranged,*—WHOLLY, ENTIRELY, ABSOLUTELY. I wish to show that these elements constitute,—not the foundation upon which, nor the materials merely with which, the science is to be subsequently constructed by some recondite and logical process of the reason,—but that they *are* the science, and the whole science, already constructed, and so far completed; and that nothing can be superadded to them by any act of the mind, which can in any way increase their value or change their character." (p. 69.)

On this we think it will be sufficient for us to point out that no such collection of facts as Dr. Bartlett regards as constituting science, can ever enable us to predict any unknown result from given data; such prediction being entirely dependent upon the discovery of the common principle connecting the phenomena, and upon the law of its operation. We find the power of making such predictions to bear an exact correspondence with the degree of generality that has been attained in the particular science, by the inductive process, which seems to Dr. Bartlett so incomprehensible, but of whose nature we shall not do our readers the injustice to suppose them ignorant.

In his eagerness to prove that every department of medical science is made up of its own distinct class of facts, Dr. Bartlett is led still further into errors, which appear to us so palpable, that we can scarcely understand how any man of his obvious ability could fall into them. Thus he asserts (p. 99) not only that "the knowledge of pathological phenomena does not *flow from* the knowledge of physiological phenomena," but also that "the science of pathology is not built upon the science of physiology." Now as every day's experience is opening new applications of physiological principles to the explanation of pathological phenomena (we could scarcely, perhaps, select a better example of this than the improved knowledge of the nature of convulsive diseases, which has resulted from the correct determination of the healthy functions of the spinal cord), we cannot believe that our readers will assent to the above-cited proposition,—any more than they will accord with others of a similar character, by which it is followed up, such as that therapeutics is not deducible from physiology or pathology. We regard these and other errors of Dr. Bartlett's as originating in the very imperfect state of these sciences at the present time; the principles which have been attained in them being of very limited generality, as well as (from the complicated nature of the phenomena to which they apply) far less certain in their operation. The very exceptions which he points out to these sweeping statements are, in our minds, but indications of the existence of the fundamental connexion which exists between the several branches of medical science. True it is that each department must have a distinct set of facts of its own; but all these facts appertain to one and the same subject,—the living human body. This body in disease is not so different from the body in health as to require an entirely new mode of considering its phenomena; on the contrary, all the knowledge which we can obtain of the changes it presents in health is so much gained towards the comprehension of the occurrences of disease. Dr. Bartlett writes as if the *facts* of physiology and pathology were as simple and palpable as those of physics or chemistry; almost entirely overlooking, as it appears to us, that nearly every one of the phenomena which commonly present themselves for observation, is made up or compounded of a number of simpler facts; and that it is frequently in the analysis of these, and in the discovery of the leading or fundamental change from which the others are deducible, that the greatest sagacity and scientific knowledge are required.

Take, for instance, almost any form of convulsive disease;—its prominent symptoms are but expressions of a disordered state of the nervous centres. This we are taught by physiology. We are further enabled, by our physiological knowledge, to limit the immediate seat of the affection to the spinal cord (the brain may, or may not, be involved in it); and we are directed to look for some *excentric* cause, which acts as the stimulus to the reflex action of the organ,—such, for example, as the irritation of teething, or of worms in the intestinal canal. Or it may be, in default of this, we shall have to look for the cause in the irritable state of the spinal cord itself; and to inquire if disordered nutrition, or retained secretions, may have perverted the condition of the blood, so as to affect this most sensitive organ.—Now it seems to us absurd to say that the facts of this pathological or morbid state are of a character altogether different from the normal or physiological phenomena which constitute the healthy function of the organ; for the *modus operandi* in each case is precisely the same; and the difference is not one of *kind* but of *degree*. Equally absurd is it to say that the therapeutic indications in such a case are not connected with the inductions of physiological science; since they are obviously and distinctly derivable from them.

In like manner, we have on a former occasion attempted to show, (vol. XVIII, p. 91), that the act of inflammation, with all its morbid phenomena, is but the result of an alteration of the natural relation between the blood and the tissues; the plasticity of the former being increased; whilst the organizing processes and general vitality of the latter are in a depressed condition. Hence this disordered state, when traced to its fundamental or original causes, is found to be much more closely related to the ordinary or normal process of nutrition, than would at first sight appear; and to depend, not on any new *kind* of property in either the solids or the fluids, but merely on an alteration in the *degree* of those, with which physiology makes us acquainted.

Though we have felt it requisite to say so much in disparagement of Dr. Bartlett's work, yet we feel bound to add, that its object is a most laudable one,—viz. to fix the attention of medical observers upon the *facts* of their science, and to lead them to collect these with truthfulness and discrimination. And whether, with Dr. Bartlett, we regard the facts as themselves constituting the science, or whether we consider them to form the basis upon which the science is to be founded, there is the same necessity for obtaining them. But it can scarcely be denied, that much of the value of medical observations depends upon the account of preliminary knowledge with which they are prosecuted. Ignorance and prejudice are alike injurious; the former leading to the neglect of many of the most important indications; the latter to the substitution of theoretical interpretations for the facts themselves. We are sorry to be compelled to believe that, taken as a class, medical men are not distinguished by their qualifications for correct observation. It was remarked long since, that there are more "false facts" than "false theories" in our science; which, considering the vast amount of the latter, is certainly giving a pretty liberal allowance to the former. Yet we are well convinced of the justice of the assertion; and shall probably make an attempt, ere long, to give a fuller expression of our own ideas on Observation and Logic in Medicine.

---

